# Severe *Plasmodium ovale* malaria complicated by acute respiratory distress syndrome in a young Caucasian man

**DOI:** 10.1186/s12936-018-2289-2

**Published:** 2018-04-02

**Authors:** Alessandra D’Abramo, Saba Gebremeskel Tekle, Marco Iannetta, Laura Scorzolini, Alessandra Oliva, Maria Grazia Paglia, Angela Corpolongo, Emanuele Nicastri

**Affiliations:** National Institute of Infectious Diseases, IRCCS, Lazzaro Spallanzani, Via Portuense 292, 00149 Rome, Italy

**Keywords:** *Plasmodium ovale*, Malaria, ARDS, Chloroquine failure

## Abstract

**Background:**

Although *Plasmodium ovale* is considered the cause of only mild malaria, a case of severe malaria due to *P. ovale* with acute respiratory distress syndrome is reported.

**Case presentation:**

A 37-year old Caucasian man returning home from Angola was admitted for ovale malaria to the National Institute for Infectious Diseases Lazzaro Spallanzani in Rome, Italy. Two days after initiation of oral chloroquine treatment, an acute respiratory distress syndrome was diagnosed through chest X-ray and chest CT scan with intravenous contrast. Intravenous artesunate and oral doxycycline were started and he made a full recovery.

**Conclusion:**

Ovale malaria is usually considered a tropical infectious disease associated with low morbidity and mortality. However, severe disease and death have occasionally been reported. In this case clinical failure of oral chloroquine treatment with clinical progression towards acute respiratory distress syndrome is described.

## Background

Although *Plasmodium ovale* is considered the cause of only mild malaria, some reports indicate the potential evolution to severe disease and even death [1]. A case of severe ovale malaria with acute respiratory distress syndrome (ARDS) unresponsive to previous therapy with chloroquine is reported.

## Case presentation

**Fig. 1:**
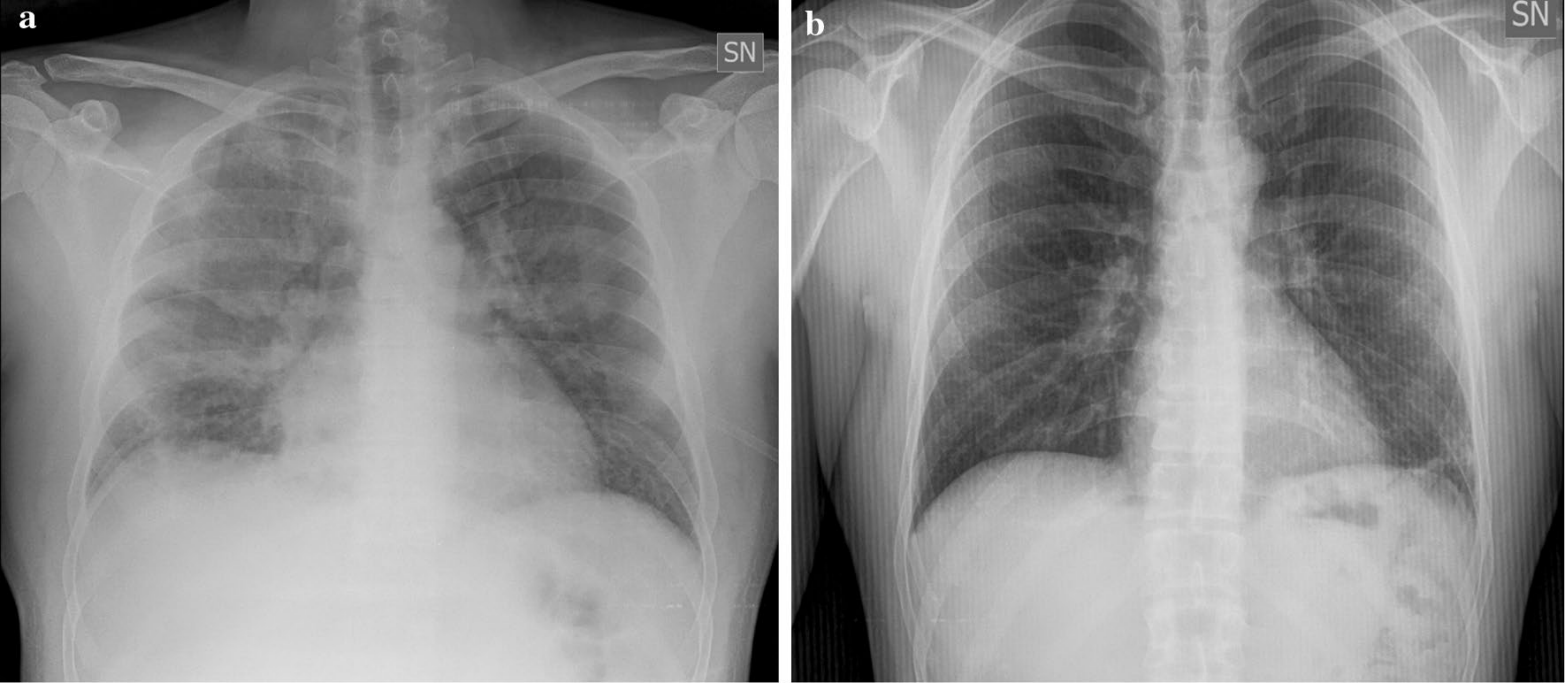
Chest X-ray before (**a**) and after (**b**) malaria *Plasmodium ovale* disease. **a** Chest X-ray showed interstitial bilateral pneumonia; **b** unremarkable results during the follow-up visit after 2 months

**Fig. 2:**
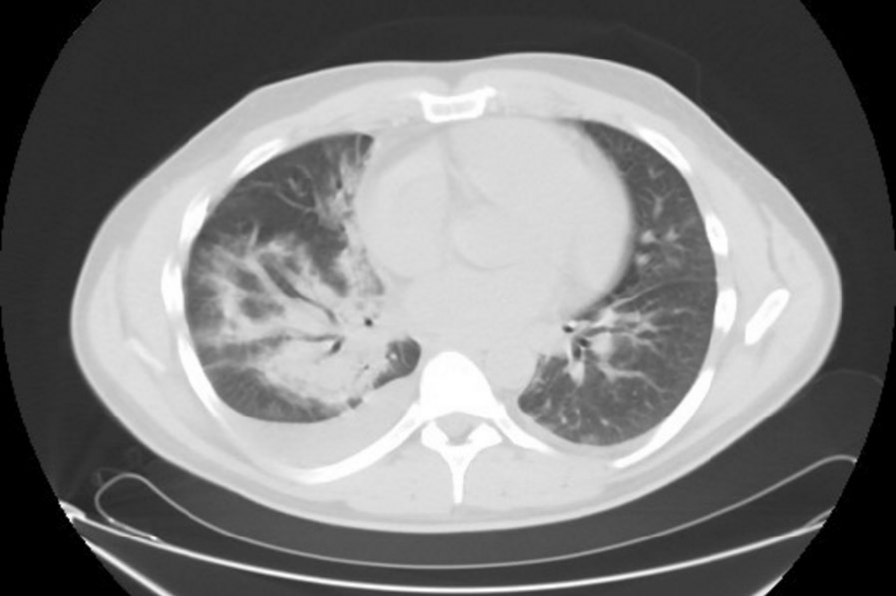
Chest computed tomography during malaria *Plasmodium ovale* disease. Chest computed tomography showed interstitial bilateral pneumonia with consensual pleural effusion

**Fig. 3:**
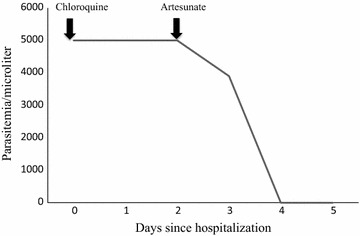
Timeline parasitemia. Parasite clearance was achieved after the introduction of intravenous artesunate

A 37-year old Caucasian man, with no co-morbidity, was admitted to the National Institute for Infectious Diseases Lazzaro Spallanzani in Rome, Italy, due to a 5-day history of fever (39 °C), headache and asthenia. Since 2013, he had been living in Angola without taking any anti-malaria chemoprophylaxis. On admission, the patient was in good condition; blood test showed only thrombocytopaenia (platelet count 63,000/mmc) with normal renal and liver function. Pan-malarial rapid test for malaria was negative while thick blood smear was positive and thin blood smear showed the presence of trophozoites and schizonts of *P. ovale*, with a 0.1% (5000/µl) parasitaemia. Oral chloroquine, 10 mg/kg as initial dose followed by 10 mg/kg on the second day and 5 mg/kg on the third day, was prescribed. In-house nested-polymerase chain reaction (PCR) confirmed the diagnosis of *P. ovale* excluding mixed infections [2]. *Plasmodium ovale wallikeri* was identified by using a nested PCR followed by 2% agarose gel electrophoresis (a 245 bp band confirmed *P. o. wallikeri*) and verified with amplicon sequencing [3]. After 2 days of well-tolerated chloroquine treatment, the patient’s condition suddenly worsened: he developed dyspnoea at rest, cough with blood-tinged sputum and high fever (39.8 °C). Chest auscultation revealed bilateral crackles in both respiratory phases. Fluid balance (input–output) was negative. Respiratory rate was 37 breaths per minute, blood oxygen saturation was 92% under oxygen supplementation with 31% fractional inspired oxygen (FiO_2_) *Venturi* mask, arterial blood gas showed an acute hypoxemia (PH: 7.45, PO_2_: 57 mmHg with an arterial oxygen tension (PaO_2_)/FiO_2_ ratio of 183) and serum albumin concentration was within normal ranges (3.9 g/dl). The patient had a persistent parasitaemia (0.1% parasitaemia) on chloroquine treatment (Fig. 3). PCR and serology for zika, chikungunya, dengue, and respiratory viruses, serology for *Leishmania* species, *Mycoplasma pneumoniae*, *Chlamydophila pneumoniae*, *Salmonella typhi* and *paratyphi*, *Rickettsia conorii* and *Treponema pallidum* were all negative; urinary antigens of *Legionella* and *Streptococcus pneumoniae* were not detectable; blood and sputum cultures were negative. Posterior-anterior chest X-ray showed bilateral infiltrates (Fig. 1a) and chest computed tomography scan with intravenous contrast confirmed interstitial ground glass opacities involving upper and inferior right and left lobes with bilateral pleural effusion and cardiomegaly (Fig. 2). Transthoracic echocardiogram showed normal heart function and ruled out cardiogenic oedema. Intravenous (iv) artesunate (2.4 mg/kg given twice daily at the first day then once daily), oral doxycycline (100 mg every 12 h), iv piperacillin/tazobactam (4.5 g every 8 h) and iv methylprednisolone (40 mg) were started. After 24-h artesunate treatment, parasite clearance was achieved and the patient’s condition improved (Fig. 3). Oral primaquine (30 mg daily for 14 days) was given. The patient was discharged in good clinical condition after 10 days of hospitalization. After 2 months, during the follow-up visit, a second chest X-ray was performed with unremarkable results (Fig. 1b) and no evidence of sequelae, relapse or haemolytic anaemia was reported.

## Discussion and conclusion

Ovale malaria is usually considered a tropical infectious disease associated with low morbidity and mortality. However, severe disease and death have previously been reported [1].

In this case, clinical failure of oral chloroquine treatment in a patient with ovale malaria is described. *Plasmodium ovale* infection was confirmed by nested-PCR targeting the small sub-unit ribosomal RNA gene, detecting at least 10 parasite genomes per reaction and mixed infection with other *Plasmodium spp* were excluded [2]. Persistent *P. ovale* parasitaemia during the first 48 h of oral chloroquine therapy was associated with clinical progression towards ARDS. Only 1 day after the switch to iv artesunate, the parasitaemia clearance was reached and the patient’s condition improved. Chloroquine is commonly used for the treatment of *P. ovale* infection. In non-falciparum malaria, resistance to chloroquine is reported only for *Plasmodium malariae* whereas *P. ovale* is usually considered fully chloroquine susceptible [4].

Moreover, in a systematic review to determine the efficacy and safety of artemisinin-based combined therapy (ACT) for the treatment of non-falciparum malaria, ACT was considered at least equivalent to chloroquine in effectively treating non-falciparum malaria [5].

Recently, 2015 WHO guidelines on malaria treatment recommend either ACT or chloroquine, with high quality of evidence in the case of non-falciparum malaria [4]. Finally, ARDS is one of the severe complications of falciparum malaria but the pathogenesis is not yet well clarified; the inflammation and the increased endothelial permeability play an important role in ARDS. Moreover, iv overhydration, increased permeability of pulmonary capillaries, sequestration of red cells, and disseminated intravascular coagulation are all other likely determinants [6]. In this case overhydration was ruled out because of negative fluid balance. As previously reported in vivax malaria, the development of respiratory distress has been associated with an inflammatory response after treatment initiation [7]. Although chloroquine is known to have an anti-inflammatory modulation, inhibiting the production of pro-inflammatory cytokines such as TNF-α, IL-1β and IL-6 [8], in this case it did not seem to prevent the inflammatory phenomenon leading to respiratory distress. In a recent review, 22 cases of severe ovale malaria were identified and in 5 of them, ARDS was reported [9]. Epidemiological and clinical features of the 5 severe ovale malaria cases complicated by ARDS are summarized in Table 1. Nevertheless, in the 2015 WHO guidelines severe ovale malaria is not mentioned and consequently diagnostic criteria and treatment indications are lacking. Further studies are needed to better define the pathogenesis of severe malaria due to *P. ovale* and the relationship between its sub-species (*wallikeri* or *curtisi*) and the clinical manifestations. Finally, the occurrence of *P. ovale* chloroquine resistance and its molecular mechanisms should be investigated.

**Table 1:** Characteristics of previous cases of ARDS in *Plasmodium ovale* malaria

Authors	Sex	Age	*P. ovale* subtype (*wallikeri/curtisi)*	Origin of infection	Time since exposition (days)	Malaria prophylaxis	Malaria naïve	Comorbidities	Parasitemia (%)	Antimalarial treatment	Invasive ventilation	Outcome	Ref
Hachimi [10]	M	31	NA	Congo	210	NA	NA	None	0.20	Quinine	Yes	Death	10
Lahlou [11]	NA	NA	NA	NA	NA	NA	NA	History of tuberculosis	0.20	Quinine	NA	Death	9
Lau [1]	M	59	Curtisi	Nigeria	180	Mefloquine	Yes	None	0.18	Chloroquine phosphate + primaquine, quinine, artesunate	Yes	Death	1
Rojo-Marcos [12]	M	43	NA	Nigeria	3	No	No	Hypertension, diabetes	0.12	Chloroquine, primaquine	Yes	Survival	11
Rozé [13]	M	24	NA	Chad ivory coast	NA	Doxycycline	NA	Tuberous sclerosis	0.20	Chloroquine, quinine	No	Survival	12

*NA* not available, *Ref* reference
